# Glycemic and Insulinemic Index Values of Apple Puree

**DOI:** 10.1002/fsn3.70844

**Published:** 2025-09-06

**Authors:** J.‐M. Lecerf, L. Moreno Aznar, L. Rjimati, F. S. Atkinson, C. Richonnet

**Affiliations:** ^1^ Institut Pasteur de Lille Lille France; ^2^ GENUD (Growth, Exercise, Nutrition and Development) Research Group Instituto Agroalimentario de Aragón (IA2) and Instituto de Investigación Sanitaria Aragón (IIS Aragón) Zaragoza Spain; ^3^ Centro de Investigación Biomédica en Red de Fisiopatología de la Obesidad y Nutrición (CIBEROBN) Instituto de Salud Carlos III Madrid Spain; ^4^ International University of Casablanca Casablanca Morocco; ^5^ Institute of Obesity, Nutrition and Exercise University of Sydney Sydney New South Wales Australia; ^6^ Materne Paris France

**Keywords:** apple puree, glycemic index, insulinemic index, processed fruits

## Abstract

A low glycemic index is known to have numerous health benefits, especially in the prevention of metabolic diseases as type 2 diabetes. The glycemic response following specific foods consumption has been reported in numerous tables, but few of them include data on apple‐based fruit desserts as puree, while they represent a major food consumed by children and adolescents. The aim of this study was to determine the glycemic and insulinemic response of two different apple purees from France and the United States, made from different raw materials and with different processes. In this cross‐over study, 10 healthy adults ingested a standard glucose solution on three separate visits before, after, and in between the test product visits and the two different apple puree in a randomized order, on one occasion only. Blood samples were collected before and all along the 2‐h after ingestion to determine the blood glucose and insulin concentrations (enzymatic assay). Consumption of the two‐apple purees led to moderate and similar glycemic and insulinemic responses. Both products presented a low glycemic index (GI) and load (GL) and insulinemic index (II). Apple purees are presenting a low GI, GL, and II and could be part of a low glycemic diet.

## Graphical abstract

A low glycemic index is known to have numerous health benefits, especially in the prevention of metabolic diseases, such as type 2 diabetes. Apple‐based fruit desserts, as puree, represent a major food consumed by children and adolescents. Using a randomized, crossover design based on the standardized international methodology for glycemic index testing (ISO 26642:2010), we demonstrate that apple purees present a low glycemic index (GI), load (GL) and insulinemic index (II).

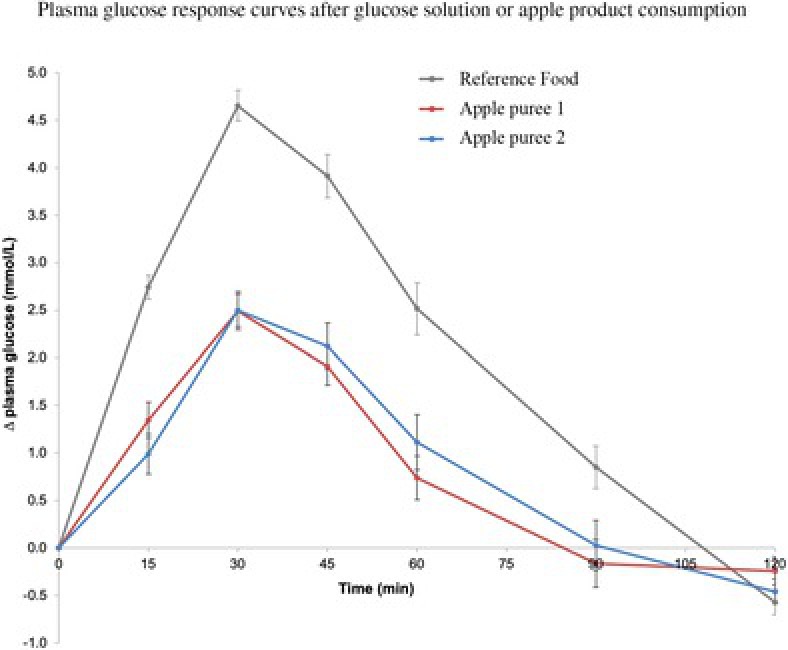

## 1 Introduction

Maintaining normal blood glucose levels and avoiding sustained hyperglycemia have been shown to induce numerous health benefits, especially in the prevention of metabolic diseases such as obesity and diabetes by limiting the postprandial glycemic response and increasing satiety (Node and Inoue 2009). Therefore, concerns about the amount and type of carbohydrates have supported the idea that some carbohydrate sources can be beneficial, while others are not, depending on both their effect on glycemia and their fiber content (Reynolds et al. 2019). The glycemic response (GR) is defined as the postprandial change in blood glucose concentration when a food or meal that contains carbohydrate is ingested (Augustin et al. 2015). The EFSA (European Food Safety Authority) considers that reducing postprandial blood glucose responses is a beneficial physiological effect as long as insulin responses are not disproportionally increased (EFSA Panel on Dietetic Products, Nutrition, and Allergies (NDA) 2010).

The glycemic index (GI) concept was introduced in 1981 by Jenkins et al. to classify the quality of carbohydrates in foods based on the extent to which they raise blood glucose levels after eating (Jenkins et al. 1981). It represents the GR elicited by a portion of food containing 50 g (or in some cases 25 g) of available carbohydrates, expressed as a percentage of the GR elicited by 50 g (or 25 g) of a reference carbohydrate, which can be a glucose solution or white wheat bread (defined as the glucose scale or bread scale, respectively). The GI ranks carbohydrates on a scale from 0 to 100. Foods with a GI above 70 on the glucose scale are considered high GI, whereas foods with a GI below 55 are considered low GI foods (Atkinson et al. 2021) (ISO 26642 2010). There are several aggregated tables of GI values for individual foods, which include published GI data from around the world (Atkinson et al. 2021, 2008; Foster‐Powell et al. 2002). However, none of these tables include GI data for any apple‐based fruit desserts as puree (Atkinson et al. 2021) while they represent a major food consumed by children and adolescents. For example, 56% of French children from 3 to 17 years old consumed on average 27.9 g per day of apple puree in 2019 (CREDOC, CCAF 2019).

Numerous dietary‐related factors contribute to the GR, including the amount and type of carbohydrates ingested, the presence of nutrients that slow gastric emptying or increase insulin secretions (e.g., fat, protein‐specific amino acids, fibers) but also the level of food processing, which may impact starch accessibility to enzymes (Augustin et al. 2015; EFSA Panel on Dietetic Products, Nutrition, and Allergies (NDA) 2010).

In apples, the main constituents that contribute to the GR and GI are carbohydrates (mainly sugars, some starch and polyols) and soluble fibers (mainly pectin). Its sugars are mainly composed of fructose and sucrose and, to a lesser extent, glucose (Atkinson et al. 2021). Those sugars do not have the same metabolic fates in the human digestive tract and impact glycemia differently. Glucose is passively transmitted into the blood system and quickly increases glycemia after ingestion. At low ingested doses, fructose is actively metabolized in the intestine into glucose and organic acids (e.g., lactate, alanine, glutamate) while the extent of passage of unmetabolized fructose through the small intestine to the liver depends on the ingested dose (Jang et al. 2018). At high doses, it seems that ingested fructose saturates the intestinal capacity for fructose metabolism (> 6 g/day, which is less than the fructose amount found in one apple) and will not cause further increases in blood glucose but may be stored as triglycerides by the liver through the de novo lipogenesis pathway (Jang et al. 2018). Therefore, fructose has a lower GI than glucose (23 vs. 100 respectively) (Merino et al. 2019). Contrary to sugars, polyols and soluble fibers have generally very low GI and tend to lower the total GI of a food (Livesey 2003). In apples, the main polyol encountered is sorbitol, which has a low GI of 9 (Livesey 2003). Apples also contain pectin, a viscous soluble fiber, which is able to reduce the postprandial GR (Muñoz‐Almagro et al. 2021).

In addition, the structure and texture, known as the “matrix effect” may influence the GI of the whole product (Haber et al. 1977; Fardet 2015). The removal of fibers from apple‐based products as juice or puree and the physical disruption of the food form can decrease satiety and alter the postprandial GR compared to the raw whole apple (Haber et al. 1977). In the Haber et al. study (Haber et al. 1977), raw apple, apple puree, and apple juice produced similar initial increases in plasma glucose; however, a more pronounced rebound decrease was reported following puree and juice consumption, but not after the whole fruit ingestion. The type of fiber present in a food and the level of physical disruption are important for the GR. The GR and the gastric emptying do not differ between refined wheat milled products and matched whole grain wheat (Pletsch et al. 2022). On the other hand, lower levels of processing or physical disruption of the starch can produce a lower GI in the case of coarse‐flour bread compared to refined‐flour bread (Yaregal et al. 2022). So, as shown in several studies (Flint et al. 2004; Dodd et al. 2011), GI is not additive, and it is not just a question of the quantity of each type of carbohydrates, fibers, or other nutrients that can impact gastric emptying which are present in a product. Then, more validated GI values for a wide range of different types of food products are needed in studies investigating effects of GI (EFSA Panel on Dietetic Products, Nutrition, and Allergies (NDA) 2010) since the final GI value of a product is not so much predictable on the basis of its nutritional composition alone.

Apart from apples as a whole, current GI tables do not present any GI data for any apple‐based fruit desserts as puree, while their consumption is increasing significantly, particularly among young people and children. In France, according to the survey of INCA 3 in 2017, 55.1% of boys and 55.7% of girls aged from 7 to 10 years consumed 46.3 and 35.7 g per day on average of apple puree, respectively. From 11 to 14 years, it is 31.5% of boys and 29.7% of girls that consumed 21.4 and 17.1 g per day of apple puree, respectively (Dubuisson et al. 2019). Therefore, the primary aim of this study was to quantify the GI of 2 different apple purees, one coming from France and the other from the United States, in order to have a vision of products made from different raw materials (different varieties of apples—bi‐colored and golden‐ whose cultivation methods differ between France and the United States) and with different processing techniques. Our hypothesis is that both no added sugar apple purees have a GI considered as low, despite the processing from the whole apple, whatever the variety of apple used as raw material and whatever the processing technique used.

## 2 Materials and Methods

This study used a randomized, crossover design based on the standardized international methodology for glycemic index testing (ISO 26642:2010). Study procedures were approved by the Human Research Ethics Committee of the University of Sydney (Protocol: 2017/801) and all participants gave written, informed consent prior to commencing the study.

### 2.1 Study Population: Characteristic and Recruitment

Ten healthy, nonsmoking adults with normal glucose tolerance and body mass index were recruited from the staff and student population of the University of Sydney. Exclusion criteria included over‐ or underweight (BMI > 25 or < 18.5 kg/m^2^); dieting or disordered eating habits; reported illness or food allergy; or regular usage of prescription medications other than standard contraceptive medication.

### 2.2 Study Design and Intervention

#### 2.2.1 Study Treatments

The food tested were two apple‐based products with no‐added sugars, the GoGo Squeeze apple puree (Materne North America, Nampa, United States) from the United States and the No added Sugar French Apple Puree (PomPote SSA) from France (Materne, Boué, France). The chemical composition of the products was analyzed by Eurofins in 2019. The GoGo Squeeze was composed of 94.2% apple, concentrated apple juice, and concentrated lemon juice. The PomPote SSA was composed of 99.7% apple puree, concentrated puree, apple natural aroma, concentrated lemon juice, and ascorbic acid. The two apple products and the reference food (glucose solution) were served to the participants in fixed test portions containing 50 g of digestible (available) carbohydrate. Glucose sugar (Glucodin powder, iNova Pharmaceuticals Aust Pty Ltd., NSW) dissolved in water was used as the reference food. The nutritional contents of the equal‐carbohydrate portions of the reference food and the two apple products are listed in Table 1 and were calculated using the manufacturer's data. Each reference beverage portion was prepared the day before required and stored overnight in the refrigerator. Each test portion of the apple products was prepared shortly before required by weighing the appropriate amount of apple purees into a standard bowl, which was then served to a participant together with a glass of 250 g of plain water.

**TABLE 1:** Weights and carbohydrates contents of the test portions of the reference food and the two test products, calculated using manufacturers' data.

Test food	Portion size (g)	Energy (kJ)	Protein (g)	Fat (g)	Total carbohydrate (g)	Available carbohydrate (g)	Total sugar (g)	Glucose (g)	Fructose (g)	Sucrose (g)	Dietary fiber (g)
Reference food (glucose sugar)	54.9 g glucose 250 g water	852	0.0	0.0	50.0	50.0	50.0	50.0	0.0	0.0	0.0
GoGo Squeez Apple Puree	346.2 g	1127	0.0	0.0	61.5	50.0	50.0	10.0	27.3	10.4	11.5
No added sugar French Apple Puree (PomPote SSA)	400.0 g	1028	1.4	2.2	55.6	50.0	45.2	8.8	28.0	9.2	5.6

#### 2.2.2 Experimental Procedures

In this cross‐over study, 10 healthy adults consumed the reference glucose solution on three separate occasions (sessions 1, 3, and 5) and the two different apple puree test foods on one occasion on the test sessions in between (sessions 2 and 4) in random order (computer‐generated randomizer program). Each session was completed on a separate morning with at least one day in between consecutive test sessions. All test sessions followed the internationally recognized methodology for GI testing, and so there was a one‐day gap between consecutive test sessions, as recommended by ISO 26642 to minimize variability in the participants' physiological responses. On the evening prior to each test session, participants also consumed a regular carbohydrate‐based meal for dinner, excluding legumes and alcohol, and then fasted for at least 10 h overnight. The next morning, participants reported to the research center in a fasting condition, and their compliance with the required preceding study conditions was confirmed by the investigators. In the fasted state (−5 and 0 min), two capillary blood samples (≥ 0.5 mL blood) were obtained from the warmed hand of a participant using a nonreusable lancet. Participants were then given a fixed portion of a test product or reference food, which they consumed with 250 g of water within 12 min. All fluid and food served, including any puree residue in the bowl, was required to be consumed. A stopwatch was started for each participant once they began eating. The participants remained at the research center for the next 2 h, during which additional blood samples were collected at 15, 30, 45, 60, 90, and 120 min after eating had commenced. Therefore, a total of eight blood samples were collected from each participant during each 2‐h test session. The participants remained seated during their test sessions, with only minimal movement permitted.

### 2.3 Measurement of Plasma Glucose and Insulin Concentrations

Capillary blood samples were centrifuged for 45 s immediately after collection. The plasma was then transferred into a labeled tube and stored at −30°C until analyzed. Plasma glucose concentrations were analyzed in duplicate using a glucose hexokinase enzymatic assay on an automatic centrifugal spectrophotometric clinical chemistry analyzer (Beckman Coulter AU480, Beckman Instruments Inc., USA). An insulin sandwich‐type enzyme‐linked immunoassay with internal standards (Insulin ELISA kit, ALPCO, Salem, NH, USA) was used to measure the insulin concentration in the plasma samples. All plasma samples were measured within the same assay.

### 2.4 Data and Statistical Analyses

The incremental area under each 2‐h plasma glucose or plasma insulin response curve (iAUC) was determined using the trapezoidal rule (ISO 26642:2010). Any area below the fasting concentration was ignored. A glycemic index (GI) and insulinemic index (II) value for each apple product was calculated for each participant by dividing their 2‐h glucose or insulin iAUC value for the test product by their average 2‐h plasma glucose or insulin iAUC value for the reference food and multiplying by 100. Similarly, the glycemic load (GL) was calculated by multiplying the amount of available carbohydrate in the portion of the food or drink by its GI value and then dividing by 100. Standard parametric statistical tests (Analysis of Variance and the LSD tests for multiple comparisons) were performed using IBM SPSS Statistics software (version 28) to determine whether there were any significant differences among the GI and II values of the two test products and the reference food. Significance was set at *p* < 0.05. Data are shown as mean ± standard error of the mean (SEM) unless otherwise stated.

## 3 Results

### 3.1 Participant Characteristics

Ten healthy adults (five males, five nonpregnant females) commenced the study and completed the required experimental test sessions. The group had a mean ± SD age of 28.5 ± 3.1 years (range: 23.9–33.4 years) and a mean ± SD body mass index (BMI) of 23.2 ± 0.7 kg/m^2^ (range: 22.3–24.9 kg/m^2^). Seven of the participants were from a Caucasian background and three were from an Asian background (two Indonesian and one Filipino).

### 3.2 Postprandial Glycemic Responses for the Reference Food and Apple Products

**FIGURE 1:**
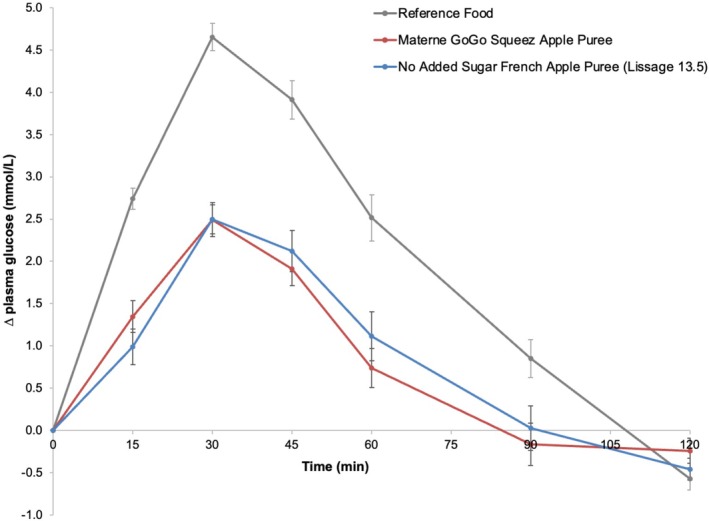
Plasma glucose response curves after glucose solution or apple product consumption. *Notes:* Mean 120‐min plasma glucose response curves in 10 healthy participants for the 50‐g available carbohydrate portions of the reference glucose solution and the two apple products, shown as the change in plasma glucose from the fasting level. Data are shown as mean ± standard error of the mean (SEM), *n* = 30 for the three repeated glucose solution tests and *n* = 10 for each of the apple products.

The absolute fasting glycemia varied between participants with an average of 4.91 ± 0.04 mmol/L across all treatments. The average 2‐h plasma glucose response curves for the 50‐g available carbohydrate portions of the reference food and the two apple products adjusted for the fasting value are shown in Figure 1. As expected, the reference food (glucose solution) produced a rapid rise in plasma glucose to a high peak concentration (4.65 ± 0.16 mmol/L adjusted for the fasting baseline level, *n* = 30) at 30 min and the greatest overall glycemic response. At 120 min, a reactive hypoglycemia occurred with a decrease of the glycemia by 0.57 mmol/L (adjusted for the baseline value) below the fasting level. Both apple samples produced a steady rise in plasma glucose concentration to a moderate peak response (2.49 ± 0.2 mmol/L for GoGo Squeez Apple Puree and 2.50 ± 0.17 mmol/L PomPote SSA adjusted for the fasting baseline level, *n* = 10 for each product) at 30 min followed by a gradual decline in glycemia between 30 and 120 min.

### 3.3 Postprandial Insulinemic Responses for the Reference Food and Apple Products

**FIGURE 2:**
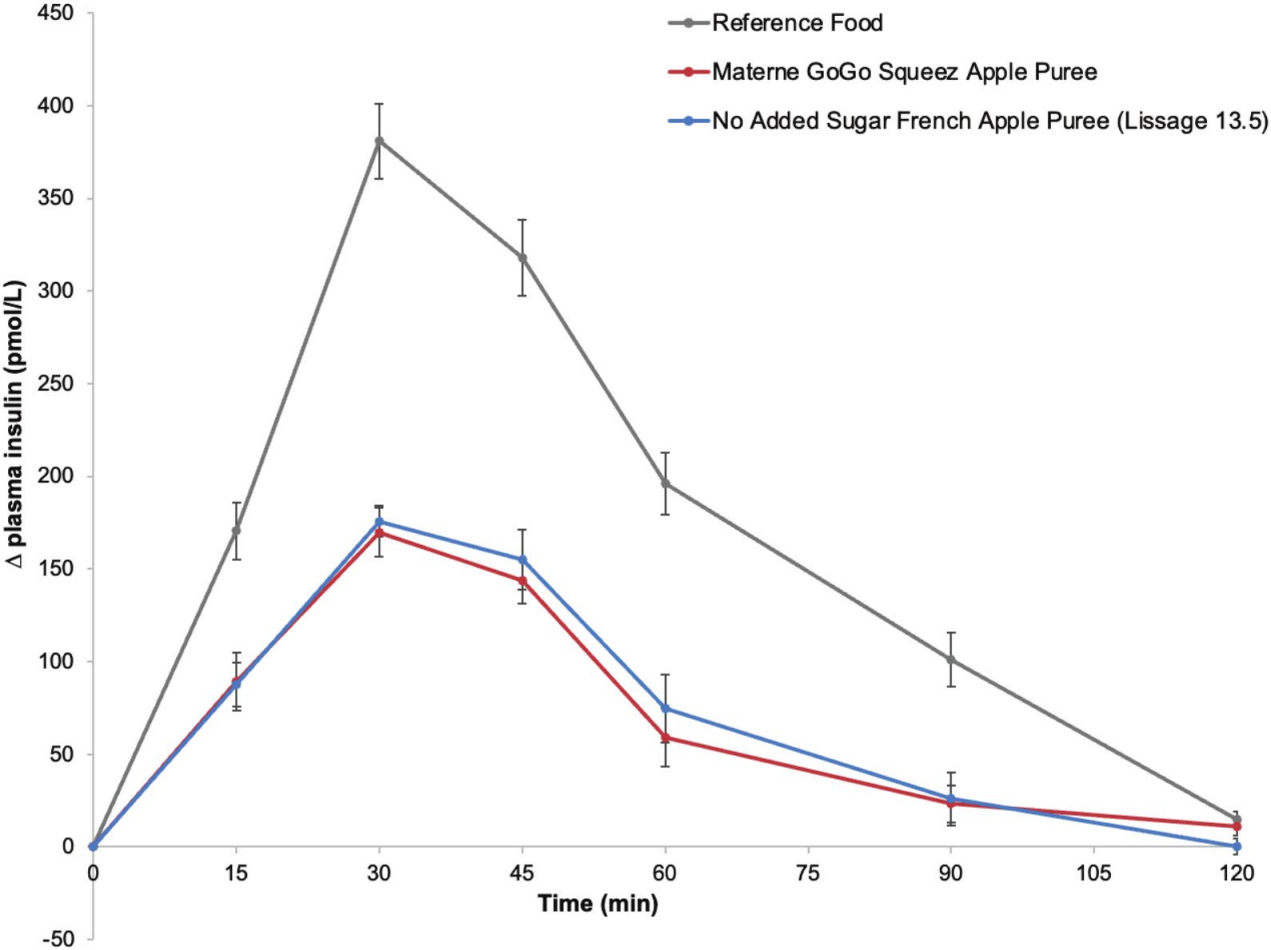
Plasma insulin response curves after glucose solution or apple product consumption. *Notes:* Mean 120‐min plasma insulin response curves in 10 healthy participants for the 50‐g available carbohydrate portions of the reference glucose solution and the two apple products, shown as the change in plasma insulin from the fasting level. Data are shown as mean ± standard error of the mean (SEM), *n* = 30 for the three repeated glucose solution tests and *n* = 10 for each of the apple products.

The absolute fasting insulinemia varied between participants with an average of 31.78 ± 0.74 pmol/L across all treatments. The average 2‐h plasma insulin response curves for the 50‐g available carbohydrate portions of the reference food and the two apple products adjusted for the fasting concentration are shown in Figure 2. As expected, the reference food (glucose solution) produced a rapid rise in plasma insulin to a high peak concentration (380 ± 20 pmol/L adjusted for the fasting level, *n* = 30) at 30 min and the greatest overall glycemic response. Both apple samples produced a steady rise in plasma insulin concentration to a moderate peak response (170 ± 13 pmol/L for GoGo Squeez Apple Puree and 176 ± 8 pmol/L PomPote SSA, adjusted for the fasting baseline level, *n* = 10 for each product) at 30 min followed by a gradual decline in insulinemia between 30 and 120 min to return to the fasting response.

### 3.4 Glycemic Index and Insulinemic Index Values

No outlier responses were observed among the GI and II values for either apple product. Therefore, the final GI value and II value for each apple product are the average of the entire group of 10 participants' data. Both tested products presented a low GI of 42 ± 3 and 46 ± 3 for the GoGo Squeez Apple Puree and the PomPote SSA, respectively. The II values were 41 ± 4 and 44 ± 3 for the GoGo Squeez Apple Puree and PomPote SSA, respectively, which were proportional to their corresponding glycemic response (II/GI = 0.96 and 0.98). The GI and II values produced by the two purees were lower than the corresponding values for the reference food (assigned GI and II value of 100, both *p* < 0.0001) but similar between the two apple products (*p* = 0.375 and 0.527, respectively). A significant positive correlation (r, p) was observed between the glucose response, the GI, and the GL.

### 3.5 Glycemic Load

Taken into account the portion consumed, the glycemic load (GL) was calculated for 90 g as the standard portion size usually found in the market. The GL of the two apple purees was 5 for a portion size of 90 g, which is still considered low (≤ 10) (Jang et al. 2018).

## 4 Discussion

Low‐glycemic diet has been shown to induce numerous health benefits, especially in the prevention of metabolic diseases such as obesity or type 2 diabetes, by limiting the postprandial glycemic response and increasing satiety (Escobedo et al. 2023; Zafar et al. 2019; Ojo et al. 2018; Basset‐Sagarminaga et al. 2023; Ni et al. 2022; Ren et al. 2023). Fruits, and notably apples, are well known for their numerous health benefits, notably through their high‐fiber content and the presence of various vitamins (e.g., vitamin C and vitamin B9), minerals (e.g., potassium, magnesium) and antioxidants (e.g., polyphenols and carotenoids) (Hyson 2011; Tian et al. 2018; Dreher 2018) but also for their content of intrinsic naturally occurring sugars. However, most of them display a low GI and GL, such as raw apples with a GI of 44 ± 5 and a GL of 7 (Atkinson et al. 2021). The glycemic response of other apple‐derived products, such as apple puree, is less well documented despite their high consumption, especially in children and young people.

However, it was shown that food processing may modify the glycemic response of the corresponding raw food as observed with the higher apple juice GI compared to the one of raw apple (Haber et al. 1977). The texture of the food is also essential to the gastric emptying time and the glycemic response (Crummett and Grosso 2022). Liquid foods lead to a quicker gastric emptying time compared to solid foods, inducing a faster glucose entrance into the small intestine and a rise in the blood glucose level (Achour et al. 2001). Therefore, the glycemic response of other apple‐derived products, such as apple puree, is essential regarding the high consumption encountered in the population, notably among youth, but is still poorly documented. This study is one of the few that has investigated the glycemic and insulinemic responses of two different apple puree products in young adults. The GI values observed for the two apple purees, which differed in their composition, are consistent with published data for raw apples (GI of 44 ± 5 and a GL of 7) and commercial infant apple puree products (commercial apple, pear and cinnamon puree from Rafferty's Graden: GI = 44 ± 4 and GL = 4; apple baby food from Czech Republic: GI = 46 ± 5 and GL = 5) (Atkinson et al. 2021).

The GoGo Squeez Apple Puree had a higher fiber amount, while the PomPote SSA had a higher protein and fat content (although weak in absolute values) with a lower simple sugar content. However, we observed no significant differences between the glycemic and insulinemic responses of these two products. Apart from the carbohydrates present, numerous food factors also influence the postprandial glycemic response of foods, such as soluble fiber, protein, fat, and polyol content (Benini et al. 1995). The presence of fat and protein in the food matrix can help to slow gastric emptying (Nuttall et al. 1984; Collier and O'Dea 1983) while soluble fiber helps to increase the viscosity of the luminal contents. Both actions can help to slow the rate and extent of glucose absorption and reduce glycemia. The higher amount of fat and protein in the PomPote SSA and the higher fiber content of the GoGo Squeez Apple Puree may have both acted to slow the metabolism of the carbohydrate in the purees to a similar extent, explaining the absence of differences in GI between the two products.

Food processing, especially disruption of the food form due to mechanical processing and/or heat, can increase the glycemic response of a starchy food (Fardet and Richonnet 2020). However, Haber and colleagues found no significant difference in acute postprandial glycemia (0–60 min) with apples processed in different ways: whole apples, apple puree, or apple juice (Haber et al. 1977). During the second hour of the experimental period, the researchers did report greater reactive hypoglycemia following the juice and puree treatments compared to the whole apple, which was attributed to higher peak serum insulin responses for the juice and puree relative to the whole apples. In our study, we did not find any significant differences in the 120‐min postprandial glucose or insulin responses between the whole Golden Delicious Apples and either puree product (data not shown). The GI values obtained for the whole Golden Delicious Apples in the present study (data not shown) were similar to the GI values for whole apples already reported in several aggregated tables of GI values for individual foods (Atkinson et al. 2021, 2008; Foster‐Powell et al. 2002; Aprea et al. 2017; Jang et al. 2018; Merino et al. 2019; Livesey 2003; Muñoz‐Almagro et al. 2021; Haber et al. 1977) and comparable to the GI measurements obtained following consumption of GoGo Squeez Apple Puree and PomPote SSA.

While numerous studies only focused on the GI, too few calculated the II and GL. Each of these indicators is complementary but cannot stand alone. The GI values are measured using portions of foods and drinks that contain either 25 or 50 g of digestible carbohydrate, but these may not be similar to the amounts of these products typically consumed by people in normal environments. The GL takes into account the portion consumed and is also useful for helping people identify which types and amounts of foods will produce relatively lower blood glucose responses after consumption. The GL of the two apple purees was 5 for a portion size of 90 g which is still considered very low (Atkinson et al. 2008).

However, apple purees are ingested four times faster than a raw apple but 4‐times less rapidly than apple juice (Haber et al. 1977). The consumption of apple purees may be less satisfying than a raw apple concerning satiety but is more satisfying than apple juices. Low glycemic foods as apple purees may induce an increase in satiety without modifying energy intake compared to higher glycemic index foods as juices or biscuits (Escobedo et al. 2023). In the specific case of youth, apple purees represent interesting products that are easy to consume with a low GI, GL, and II compared to apple juice or traditional biscuits, notably served as snacks. Furthermore, the applicability of the GI to mixed meals is not always relevant as the GR of a mixed meal is not equivalent to the sum of each food GI (Dodd et al. 2011). In fact, the GR and the GI of a whole meal are more strongly correlated to the total energy, fat, and protein contents than to carbohydrate content alone (Flint et al. 2004) while simply the order of food ingestion may also modify or attenuate the postprandial glycemic response to a meal (Wu et al. 2022). Thus, apple purees may be good low GI and II food products to incorporate into a healthy low‐glycemic index diet.

Despite the interesting results, our study was conducted in young adults while the highest consumers are children and adolescents. Nevertheless, this allows for comparison with other studies and other food products, as the majority were performed in adults. Furthermore, there is no direct comparison in this study with GI and II of raw apple. It would be also interesting to test apple puree made with other varieties of apples. Further studies are needed investigating the GI and II of different types of apple products in children and adolescents specifically. Beyond the traditional GI, GL, and II for carbohydrate quality assessment, new ratio‐based carbohydrate quality metrics are emerging, basing their evaluation on overall nutritional quality through total carbohydrates‐fibers‐free sugars ratios and higher levels of health‐promoting nutrients such as protein, dietary fiber, vitamins, and minerals (Tan et al. 2023). With such metrics, apple purees may also be well classified as poorly processed and high‐fiber‐content products. Future studies should also investigate the carbohydrate quality of apple puree using these emerging metrics.

## 5 Conclusions

Preserving a normal blood glucose level and avoiding glycemia peaks are essential for long‐term metabolic health. Apple purees are consumed by all age groups around the world and especially among children and adolescents. Despite the processing, apple puree induces a low GI and II response not different from a raw apple in young adults. Apple purees may represent a good alternative to traditional high‐sugar desserts, such as cakes and milky desserts. However, more studies on apple puree are needed, focusing on children and adolescents and evaluating carbohydrate quality through more reliable and new metrics considering the degree of processing as well as the fiber and micronutrient contents.

## Author Contributions

**J.‐M. Lecerf:** methodology (equal), supervision (lead), writing – review and editing (equal). **L. Rjimati:** validation (equal), writing – review and editing (equal). **F. S. Atkinson:** conceptualization (equal), formal analysis (equal), methodology (equal), project administration (equal), writing – review and editing (equal). **C. Richonnet:** conceptualization (equal), data curation (equal), writing – original draft (lead).

## Ethics Statement

Ethical Approval Study procedures were approved by the Human Research Ethics Committee of the University of Sydney (Protocol: 2017/801).

## Consent

All the authors consented to the publication of this article.

## Data Availability

The datasets in this study are available from the corresponding author on reasonable request.
